# Aligning the Global Delta Risk Index with SDG and SFDRR global frameworks to assess risk to socio-ecological systems in river deltas

**DOI:** 10.1007/s11625-023-01295-3

**Published:** 2023-03-03

**Authors:** Emilie Cremin, Jack O’Connor, Sumana Banerjee, Ly Ha Bui, Abhra Chanda, Hieu Hong Hua, Da Van Huynh, Hue Le, Sonia Binte Murshed, Salehin Mashfiqus, Anh Vu, Zita Sebesvari, Andy Large, Fabrice G. Renaud

**Affiliations:** 1 School of Interdisciplinary Studies, The University of Glasgow, Dumfries Campus, Rutherford/McCowan Building, Crichton University Campus, DG1 4ZL Dumfries, Scotland, UK; 2 United Nations University, Bonn, Germany; 3 Jadavpur University, Kolkata, India; 4 CRES, Vietnam National University, Ho Chi Minh City, Vietnam; 5 Can Tho University, Can Tho, Vietnam; 6 Institute of Water and Flood Management, Bangladesh University of Engineering and Technology, Dhaka, Bangladesh; 7 NatCen International, National Centre for Social Research, London, UK; 8 School of Geography, Politics and Sociology, Newcastle University, Newcastle, UK

**Keywords:** Risk, Vulnerability, Exposure, SDGs, Sendai Framework for Disaster Risk Reduction, River deltas, Framework integration

## Abstract

River deltas globally are highly exposed and vulnerable to natural hazards and are often over-exploited landforms. The Global Delta Risk Index (GDRI) was developed to assess multi-hazard risk in river deltas and support decision-making in risk reduction interventions in delta regions. Disasters have significant impacts on the progress towards the Sustainable Development Goals (SDGs). However, despite the strong interlinkage between disaster risk reduction and sustainable development, global frameworks are still developed in isolation and actions to address them are delegated to different institutions. Greater alignment between frameworks would both simplify monitoring progress towards disaster risk reduction and sustainable development and increase capacity to address data gaps in relation to indicator-based assessments for both processes. This research aims at aligning the GDRI indicators with the SDGs and the Sendai Framework for Disaster and Risk Reduction (SFDRR). While the GDRI has a modular indicator library, the most relevant indicators for this research were selected through a delta-specific impact chain designed in consultation with experts, communities and stakeholders in three delta regions: the Red River and Mekong deltas in Vietnam and the Ganges–Brahmaputra–Meghna (GBM) delta in Bangladesh and India. We analyse how effectively the 143 indicators for the GDRI match (or not) the SDG and SFDRR global frameworks. We demonstrate the interconnections of the different drivers of risk to better inform risk management and in turn support delta-level interventions towards improved sustainability and resilience of these Asian mega-deltas.

**Supplementary Information:**

The online version contains supplementary material available at 10.1007/s11625-023-01295-3.

## Introduction 

River deltas, formed by the deposition of sediments brought from upstream, are fertile landscapes constantly reshaped by the forces of river waters and tides (Bianchi and Allison 2009; Nicholls et al. 2020). Despite being regions of high agricultural productivity, river delta socio-ecological systems (SESs) are also exposed to multiple natural hazards, including land subsidence, riverine and coastal floods, coastal and river erosion, and cyclones and storm surges (Syvitski et al. 2009; Anthony et al. 2015; Brown and Nicholls 2015; Haque and Nicholls 2018). This dynamism puts pressure on local livelihoods (Smith et al. 2013; Renaud et al. 2016; Hossain et al. 2018). The impacts of climate change (IPCC 2019; Reisinger et al. 2020; Das et al. 2020b; Masson-Delmotte et al. 2021) and related increases in hazards’ frequencies and magnitudes, as well as social and ecological vulnerability* (e.g. malnutrition, poverty and unequal access to services between rural and urban areas), continue to challenge the achievement of the Sustainable Development Goals (SDGs) for deltas (Szabo et al. 2016b; Adams et al. 2018, 2020; Hutton et al. 2018; Renaud et al. 2022).

While economies in delta regions have been growing with the expansion of aquaculture, disasters* have significant impacts on the implementation and progress of the SDGs as well as through their occurrence underlining the importance of more effective disaster risk reduction action in underpinning sustainable development (UNDRR 2015a, b; Chmutina et al. 2021). Moreover, any ‘deficit’ in sustainable development increases vulnerability and becomes a multiplier for disaster risk (Dazé et al. 2018; Flood et al. 2022).

To address risk reduction challenges, in the context of accelerating climate change, the United Nations invite member states to prepare national adaptation plans (NAPs) and to implement policies in line with the Paris Agreement on climate change and the Sendai Framework for Disaster Risk Reduction (SFDRR) (UNDRR 2020).

Synergies and trade-offs are already identified within the SDG framework (Kroll et al. 2019; Hegre et al. 2020; Renaud et al. 2022) due to the interconnected nature of climate change, biodiversity, disaster risk and sustainable development (Scharlemann et al. 2020).

Similar links exist between global frameworks, but goals and targets remain siloed within each framework, increasing monitoring and reporting efforts for member states (OECD 2020). Engaging with global frameworks separately can, therefore, present a somewhat overwhelming agenda for stakeholders and governments. Any mismatch between overarching frameworks can hinder actions, slow progress and potentially have negative impacts on management efforts at regional, national and sub-national levels.

Given the complexity of the interlinkages between different goals, targets and indicators, there is a need to better understand how the vulnerability of social–ecological systems aligns with, and is influenced by, global policies. To address this gap, we argue that the assessment of disaster risk would be greatly enhanced by the integration of SDGs data in future iterations of disaster risk reduction frameworks for action; see also Chmutina et al. (2021). Multi-hazard* risk assessments have been developed based on different frameworks and progress in disaster and risk sciences (Gallopín 2006; Cheung 2007; Renaud et al. 2010; Birkmann et al. 2012). These are applied to the global or the regional scales (Marin-Ferrer et al. 2017; Hill et al. 2020; Das et al. 2021) and help to assess the implementation of the SFDRR (UNDRR 2020).

The Global Delta Risk Index (GDRI) offers a comprehensive framework and a tool to assess risk to SESs in deltas both in terms of irreversible changes—including tipping points—and the decline of human well-being and sustainable livelihoods (Sebesvari et al. 2016; Hagenlocher et al. 2018a; Anderson et al. 2021). The GDRI seeks to spatially analyse different components of social–ecological risk at the scale of sub-delta administrative units, thereby enabling cross-delta and inter-delta comparisons. This index captures not only hazards and exposure, and ecological and social susceptibilities, but also information on ecological robustness and on coping and adaptation capacities to reduce/minimise social–ecological vulnerability.

Risk to SESs can be assessed both through qualitative and quantitative modelling approaches and many different methodologies and tools have been developed and tested to analyse interactions between different ecological and social systems (Voinov and Bousquet 2010; Voinov et al. 2018). Used as a qualitative modelling tool, impact chains are a useful analytical tool that helps in understanding and prioritising the factors that drive risk in the system of concern. This qualitative model shows the relations between different processes or variables that can be further measured by indicators (Hagenlocher et al. 2018b; Zebisch et al. 2021).

Here, we present the process and the result of a targeted consultation, engaging stakeholders in the co-production of impact chains to enable the selection of indicators for three major mega-deltas in South and Southeast Asia: the Red River (RRD) and the Mekong River (MRD) deltas in Vietnam and the transboundary Ganges–Brahmaputra–Meghna delta in Bangladesh (GBM-B) and India (GBM-I). These deltas are selected as they are globally significant, encompass a range of biophysical and social conditions and are under rising threat from anthropogenic stressors and climate change. All three deltas are the specific focus of the transdisciplinary UKRI GCRF Living Deltas Hub^1^ through which this research is carried out.

We present the outcome of the consultation leading to the selection of the relevant indicators from two global frameworks: the UN SDGs and the SFDRR. We then analyse how effectively the 143 indicators of the GDRI modular library match (or not) the SDG and SFDRR global frameworks.

The aim of this research is to support the implementation of better-informed policies to address risk to livelihoods in these mega-deltas. Such risk assessments are essential to (a) highlight the most important drivers of change threatening community livelihoods and (b) to illustrate shortcomings of policies for the implementation of the SDGs, so that policies can be improved for the future.

## Methods

### GDRI conceptual reframing and derived impact chain tool

Impact chains are a valuable tool for visualising cascading effects of multiple hazards, changes produced by anthropogenic drivers and the vulnerability of socio-ecological systems. In our context, impact chains fully incorporate information on delta communities’ livelihoods and assets and risk to both. Each component integrates multiple interacting sub-components in a web of interconnected factors, translated into indicators (including a selection of SDGs). The flow can incorporate linear cause–effect chains as well as feedback loops (Hagenlocher et al. 2018b; Zebisch et al. 2021; Yuen et al. 2021).

Based on the GDRI conceptual framework, we used impact chains to integrate empirical knowledge and to analyse the complex social–ecological systems (Hagenlocher et al. 2018b; Zebisch et al. 2021). This is achieved by including and linking the components of risk with the SDGs and SFDRR indicators. The GDRI embodies multiple spatial dimensions: global atmospheric and geophysical dynamics, whole river basin processes and drivers and those at the delta, the sub-delta and the local scales. The approach takes into consideration the governance systems that influence economic and environmental agendas from the international level down to the local level.

**Fig. 1:**
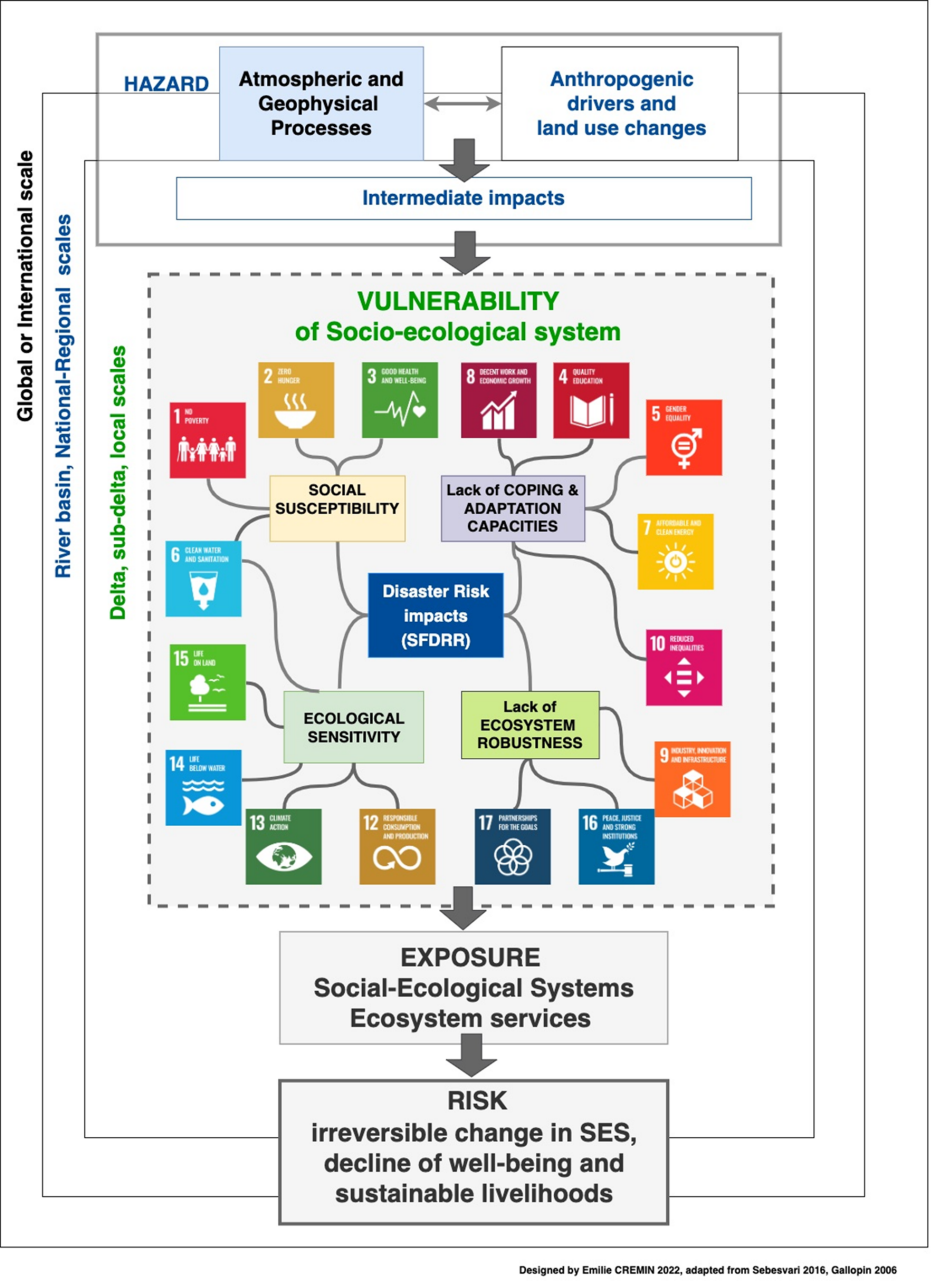
Alignment of the Global Delta Risk Index original framework with the Sendai Framework for Disaster Risk Reduction and Sustainable Development Goals. Source: adapted from Sebesvari et al. (2016)

Hazards are considered here as single and/or combined atmospheric and geophysical processes interconnected with anthropogenic drivers of land use changes and the consequent cascading and compounding effects, leading to intermediate impacts (Fig. 1). Right from the outset, we could already observe the links between SDGs and SFDRR targets and the vulnerability components of the GDRI: social susceptibility* can integrate indicators of the SDG 1 No Poverty, SDG 2 Zero Hunger, SDG 3 Good Health and Well-being, SDG 6 Clean Water and Sanitation, SDG 10 Reduced Inequalities, SDG 11 Sustainable Cities and Communities; the lack of coping and adaptation capacities* can be aligned to SDG 4 Quality Education, SDG 5 Gender Equality, SDG 7 Affordable and Clean Energy, SDG 8 Decent Work and Economic Growth, SDG 9 Industry, Innovation and Infrastructure; ecological sensitivity* can be linked to SDG 11 Sustainable Cities and Communities, SDG 12 Responsible Consumption and Production; the lack of ecological robustness* can be linked to SDG 13 Climate Action, SDG 14 Life Below Water, SDG 15 Biodiversity, SDG 16 Peace, Justice and Strong Institutions, SDG 17 Partnerships to achieve the Goals (for more details on these concepts see Supplementary Material 1—S1). Similarly, the SFDRR targets and indicators can be deployed within the components of vulnerability and we bundle them within the disaster risk impacts in the centre of Fig. 1. It is clear that SDG and SFDRR targets and goals can be integrated into multiple components of the risk. We clarify and detail these links through the overall methodological process.

In this reframed version of the GDRI (Fig. 1), we integrate and align a selection of delta-specific indicators with the indicators used for the assessment of the SDG and SFDRR global frameworks to better assess the vulnerability of SESs in the river deltas. This helps improve the geographical and cultural relevance of the index-based assessments and therefore aids formal monitoring and reporting to the international arena (e.g. national Voluntary National Reviews). This figure only captures a few links between the components of the GDRI, SDGs and SFDRR. However, more interlinkages were identified through a consultation leading to the design of the impact chain (S3).

### Stakeholder engagement and support from scientific evidence

Impact chains were developed through consultations with experts and stakeholders in our three delta SESs. The expert groups were multidisciplinary team of scientists in social and environmental sciences with specialist knowledge for each of our targeted river deltas. The experts joined together to form a wider Risk Assessment Working Group (RAWG) between October 2020 and April 2022. The members of the group, co-authors of this paper, have revised the GDRI conceptual framework with the SDG and the SFDRR frameworks (Fig. 1). The conceptual framework was used as the background for the first version of the impact chain, drafted by our team, based on each expert's knowledge of processes taking place in deltas and complemented with a non-systematic literature review.

In the next step, delta teams oversaw the design of a series of more SES-specific impact chains for each of their delta regions. This was achieved through key informant interviews (KII), focus group discussions (FGD) with delta communities (e.g. groups of people with a shared identity or interest that have the capacity to act or express itself as a collective), and/or workshops with a group of stakeholders (e.g. elected members of the local communities, government officers, managers of industry, representatives of non-governmental organisations and others) (Table 1).

**Table 1:** Sources of information for the impact chain

Delta	Number of key informant interviews	Number of participants in focus group discussions	Number of participants in stakeholder workshops	*M* %	*F* %	*M* (*n*)	*F* (*n*)	Total
Ganges Brahmaputra Meghna, India (GBM-I)	14	0	0	50	50	7	7	14
Ganges Brahmaputra Meghna, Bangladesh (GBM-B)	0	56	10	82	18	54	12	66
Red River Delta, Vietnam (RRD)	78	80	0	56	44	89	69	158
Mekong River Delta, Vietnam (MRD)	0	59	31	76	24	68	22	90

The delta teams had different constraints (including COVID-19 or other administrative restrictions) when organising activities; therefore, the type of activity and levels of public engagement differed from delta to delta. During the consultation process, stakeholders and communities were invited to revise a draft of the delta-specific impact chains designed by the experts or interviewed through an open-ended questionnaire to design delta-specific impact chains. Several questions were designed to understand the root causes of risk. Participants were asked to identify (i) drivers of change and threats faced by the ecological and social sub-systems, (ii) differentiated threats for both systems (social and ecological), if different, (iii) key components for the social susceptibility and the adaptation and coping capacities, (iv) key components for the ecological sensitivity and the potential robustness and (v) the elements exposed to hazard, human, or ecological spaces.

Country-level ethical clearance was secured by national lead organisations for the key informant interviews, the focus group discussion and the stakeholder workshops and the overall approach received ethical clearance from the College of Social Sciences of the University of Glasgow.

### Literature review to support the arguments of the stakeholders

The facts and the arguments collected during stakeholder engagement activities are all supported by scientific evidence. Therefore, before and after the consultation, we confirmed all the arguments with an online literature review based on Google Scholar and Web of Science using search terms linked to environmental risk (“hazard”, “exposure”, “vulnerability”, “social–ecological systems”, “Mekong”, “GBM”, “Red River”, “delta”) in the GBM-I, GBM-B, RRD and/or MRD. In addition, we reviewed the bibliographies of the reviewed papers and scientific reports from UN organisations and non-governmental organisations to follow up with any other relevant literature that was not listed in our search. All the results presented in this paper are underpinned by the evidence from this literature review.

### Development of the indicator list and alignment with global frameworks

Once all the delta-specific impact chains were developed, a final workshop was organised with delta experts to merge all the information in one impact chain presented in this paper (see: Supplementary Material 3—S3). Creating a generic impact chain allowed synthesis of the analysis and quantification of the correspondence between the components of the GDRI and the targets of the SDGs (S4). Second, a list of indicators was generated based on the impact chain to compare conditions across the river deltas. These were integrated into the indicator library of the GDRI, where they are classified by components and categories (see S4). The library composition is modular, meaning that the content can be adapted to a river delta context and specificities. Following this, the updated list of indicators was further analysed by the expert team to derive a comparison between the SDGs and SFDRR indicators. Where indicators were seen to be identical, they were combined as one. The result of this process is summarised in two matrices (Tables 2 and 3). These show the number of indicators selected that are identical between frameworks and so the alignment between the different components of the GDRI and the targets of the SDGs and the SFDRR.

**Table 2:** SDG/GDRI matrix presenting the number of indicators selected for the GDRI per SDG

SDG	GDRI						
	Social susceptibility	Coping and adaptation	Ecosystem robustness	Ecosystem sensitivity	Ecosystem exposure	Social exposure	Total
1. No poverty	6	4				2	**12**
2. Zero hunger	5	5					**10**
3. Good health and well-being		3				2	**5**
4. Quality education	3	2					**5**
5. Gender equality	2						**2**
6. Clean water and sanitation	3	1	3				**7**
7. Affordable and clean energy	1						**1**
8. Decent work and economic growth	2						**2**
9. Industry, innovation and infrastructures	1	3					**4**
10. Reduced inequalities	2	1					**3**
11. Sustainable cities	2	3				2	**7**
12. Sustainable consumption and production		1					**1**
13. Climate action		4				1	**5**
14. Life below water	1		1	2			**4**
15. Life on land			6	4			**10**
16. Peace, justice and strong institutions	1	1					**2**
17. Partnership for the goals		2					**2**
Total	**29**	**30**	**10**	**6**	**0**	**7**	**82**

Bold is used to highlight the results

**Table 3:** GDRI/SFDRR matrix presenting the number of indicators selected for the GDRI

SFDRR	GDRI						
	Social susceptibility	Lack of coping and adaptation strategies	Ecosystem robustness	Ecosystem sensitivity	Ecosystem exposure	Social exposure	Total
A. Reduce global disaster mortality						3	**3**
B. Reduce the number of affected people						5	**5**
C. Reduce direct disaster economic loss	2			1		2	**5**
D. Reduce disaster damage to critical infrastructure and disruption of basic services (health and educational facilities)		2				2	**4**
E. Increase the number of countries with national and local disaster risk reduction strategies		3					**3**
F. Enhance international cooperation to developing countries		2					**2**
G. Increase the availability of and access to multi-hazard early warning systems and disaster risk information	3	3					**6**
Total	**5**	**10**	**0**	**1**	**0**	**12**	**28**

Bold is used to highlight the results

The methodology presented here represents stages 1 and 2 of the overall risk assessment work plan (overall, 7 stages are presented in S2) to produce comprehensive environmental risk assessments at the delta scale and at the local coastal scale to support policy makers in the planning of delta policies.

## Results: risk and sustainability and linking vulnerability indicators with the SDGs and SFDRR

### Cascading effects and impacts of natural hazards and exposure

**Fig. 2:**
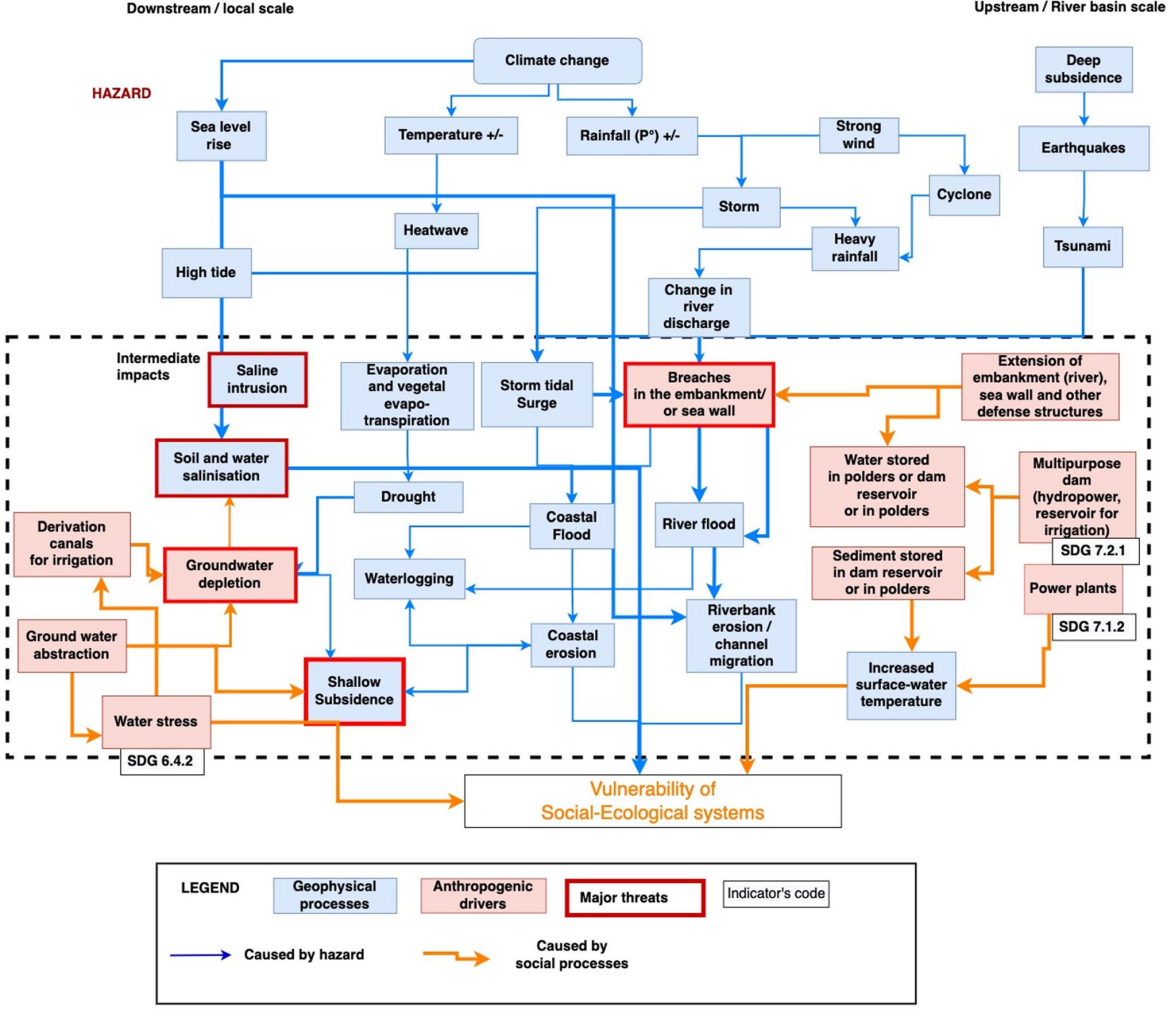
Hazard and intermediate impacts, including anthropogenic drivers of change. All the geophysical processes have cascading effects and impacts on the vulnerability of social–ecological systems, with reference to SDG indicators (extract of the impact chain S3). Source: Impact chain designed through consultation with UKRI GCRF Living delta team and participants to stakeholders’ workshops in the Red River Delta, the Mekong River Delta and Ganges–Meghna–Brahmaputra deltas in India and Bangladesh. Edited by Emilie Cremin, 2022

In the GDRI library, the hazards resulting from hydro-climatological (climate-driven rainfall and temperature rise; sea-level rise) and geophysical (deep subsidence; earthquakes; tsunamis) processes can have cascading effects. Neither SDGs nor SFDRR indicators measure natural hazards, but a selection of SDG indicators can measure the impact of anthropogenic drivers (Fig. 2).

#### Impact of cyclones, storm surges, sea-level rise, high tides and tidal floods

In all three river mega-deltas, experts, stakeholders and communities have reported that cyclones and storms bring intense rainfall and strong winds that damage properties and endanger lives and livelihoods. The impacts of super cyclones Amphan and Yaas on the GBM-I and GBM-B in May 2020 and 2021, respectively, were reported by the stakeholders as major events in recent years. During both cyclones, winds were so strong that sections of the mangroves shielding the coast were uprooted (Mishra et al. 2021, 2022) and agricultural fields were destroyed (Ali et al. 2020). More than 11,000 km^2^ of land was flooded in the GBM-I (Halder and Bandyopadhyay 2022). In all three deltas, heavy rainfall during the monsoon season may also lead to salinity intrusion (Saha 2017) and rises in water discharge, leading to river flooding and increased riverbank erosion. Sea-level rise is already increasing pressures on the coast as tides are becoming higher. Storm surges and tidal floods were reported as a main cause of coastal flooding and the main accelerator of coastal erosion in all the deltas, especially in the Mekong River delta (Fig. 2).

#### Impact of geophysical dynamics

As the GBM and the RRD are located along fault lines and, thus, potentially on seismic zones, deep subsidence can involve earthquakes and tsunami (Alam 2019; Hossain et al. 2020b). However, the tsunami event of December 2004 (Ioualalen et al. 2007) generated relatively minor impacts across the north-eastern Indian coast when compared to impacts in Indonesia, Sri Lanka and Thailand.

#### Saline intrusion and increased soil and water salinity

The compound effects of storm surges and sea-level rise lead to increases in saline intrusion resulting in soil (Durand et al. 2011; Szabo et al. 2016a; Abdullah et al. 2019; Nguyen et al. 2019; Das et al. 2020a; Hassani et al. 2021) and freshwater salinisation (Nishat and Mukherjee 2013; van Tho 2022). Heatwaves increase evapotranspiration and consequently drought and groundwater depletion during the dry season, as groundwater cannot be recharged (Alauddin and Sarker 2014; Mukherjee et al. 2018; Das and Mukherjee 2019; Hossain et al. 2020a; Fig. 2).

### Compounding effects

#### Impact of dams, hydraulic infrastructures and power plants

**Fig. 3:**
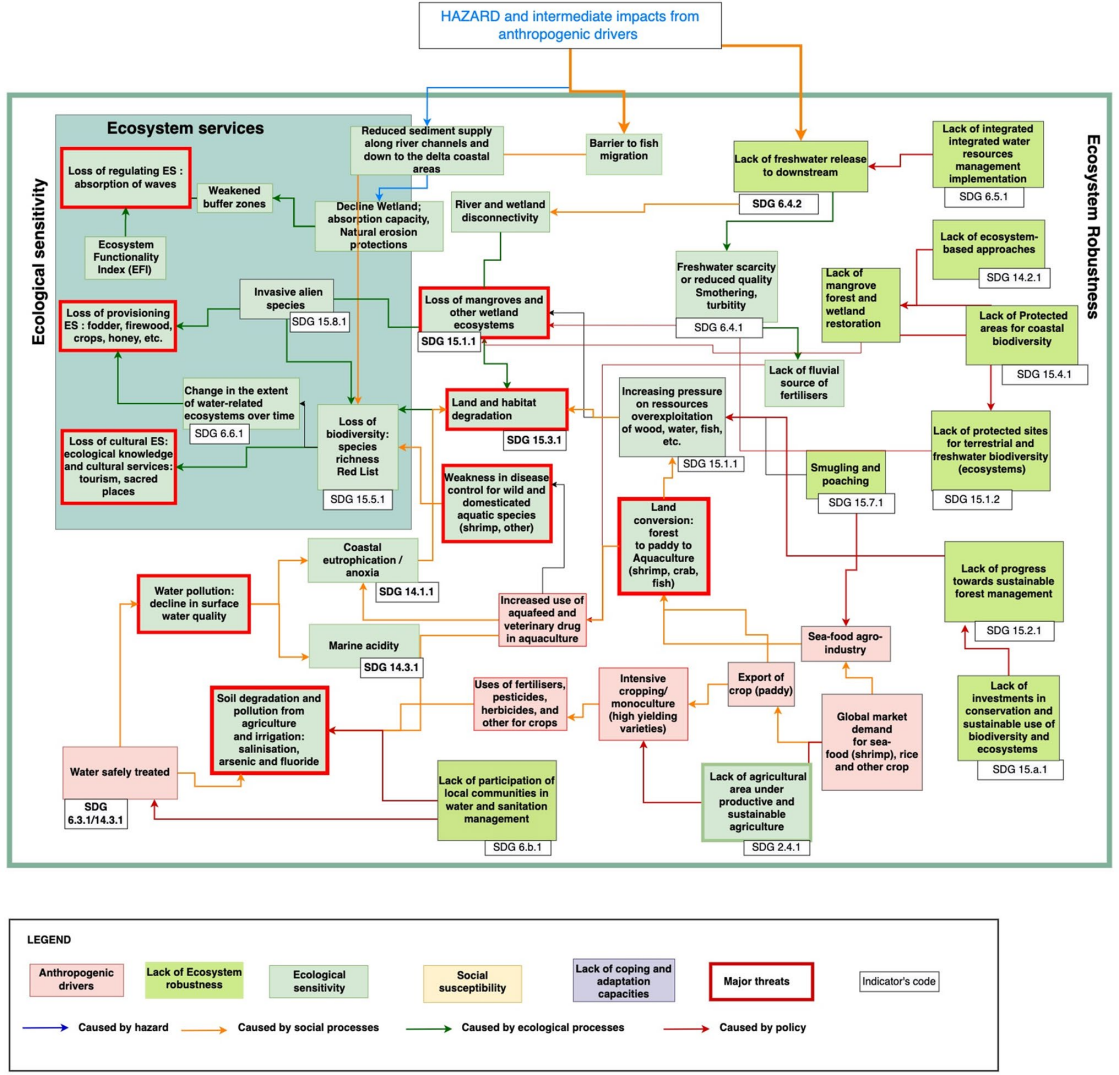
Ecological vulnerability: result of ecological sensitivity, the lack of ecosystem robustness and the impacts of hazards and intermediate impacts from anthropogenic drivers, with reference to SDG indicators (extract of the Impact chain S3). Source: Impact chain designed through consultation with UKRI GCRF Living delta team and participants to stakeholders’ workshops in the Red River Delta, the Mekong River Delta and Ganges–Meghna–Brahmaputra deltas in India and Bangladesh. Edited by Emilie Cremin, 2022

**Fig. 4:**
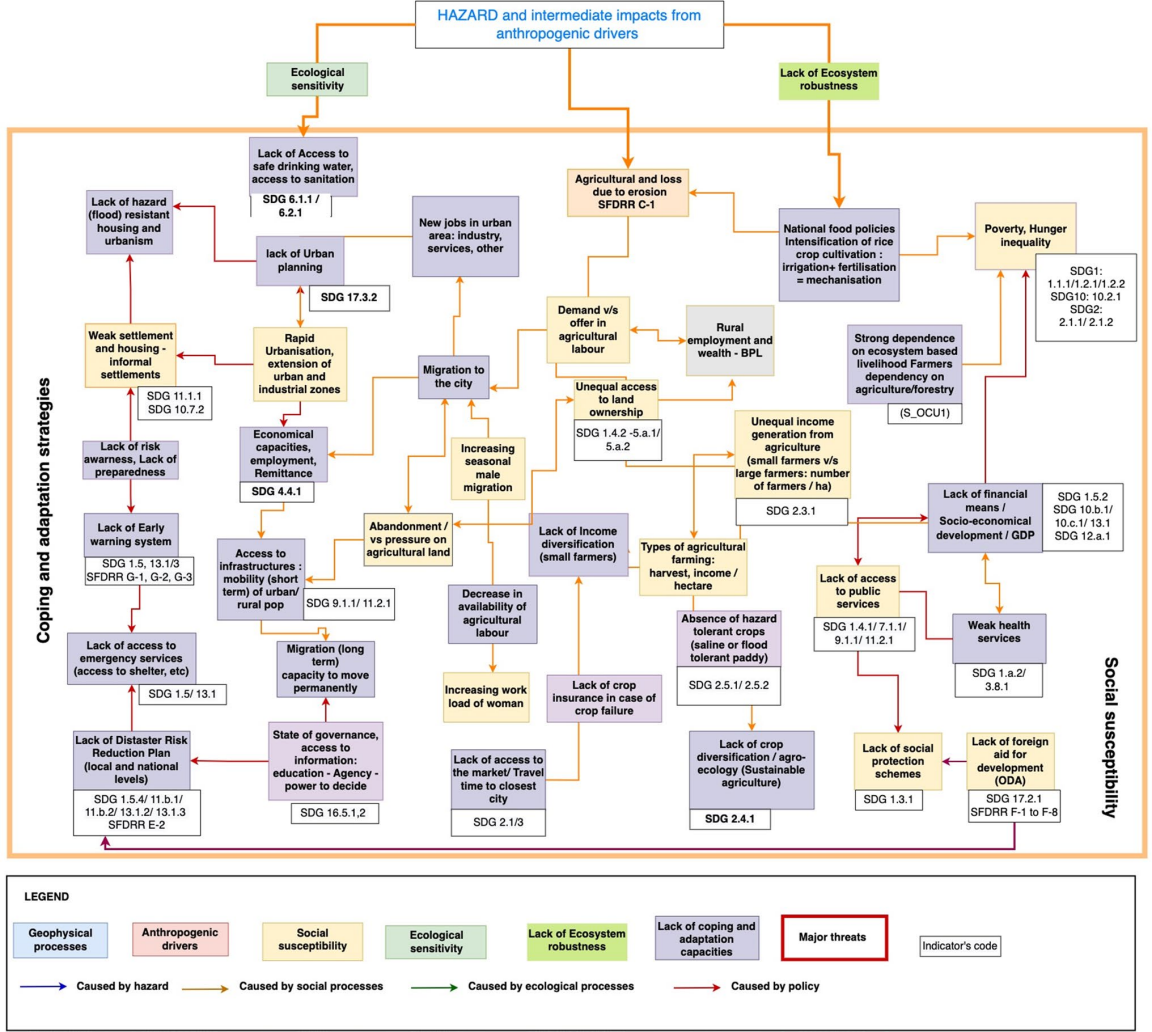
Social vulnerability: coping and adaptation strategies and social susceptibility, with reference to SDGs and SFDRR indicators (extract of the impact chain S3). Source: Impact chain designed through consultation with UKRI GCRF Living delta team and participants to stakeholders’ workshops in the Red River Delta, the Mekong River Delta and Ganges–Meghna–Brahmaputra deltas in India and Bangladesh. Edited by Emilie Cremin, 2022

Multipurpose dams (for hydropower and/or reservoir for irrigation) of different heights (10–260 m) have been constructed in the Red River, in the Mekong and in the Ganges–Brahmaputra River basins^2^ (Molle et al. 2012; Kuenzer et al. 2013; Murshed et al. 2019). The production of energy is in line with SDG7 “Affordable and clean energy”, with targets on increasing access to electricity and the share of renewable energy used to produce it (SDG 7.1.2; 7.2.1). However, the infrastructures used to produce energy (dams or power plants) can create trade-offs with other SDGs including the SDG 14—Life below water and SDG 15—Life on Earth. For example, dams and power plants can increase the temperature of water, change the quality of water and restrict fish migration within the entire river and coastal system, leading to a loss of biodiversity (Blaikie and Muldavin 2004; Hossain et al. 2018; Ahmed et al. 2018) (Fig. 2). Dams, hydraulic infrastructures and power plants have further impacts on the “ecological robustness” and the “ecological sensitivity” (Fig. 3 and Fig. 4).

#### Shallow subsidence

The subsidence is exacerbated by the retention of sediments upstream in reservoirs and by the underground water extraction in the deltas themselves. The groundwater abstraction leads to increased salinity and arsenic contamination in groundwater (Ahmad et al. 2018; Bai et al. 2019) and contributes to the subsidence of the deltas with the effect generally being most visible in urban areas (Becker et al. 2020). Measuring the extent of land subsidence is highly relevant for quantifying the vulnerability of deltas to the combined effects of flooding along with sea-level rise (Syvitski 2008; Syvitski et al. 2009).

### Social–ecological vulnerability

#### Ecological sensitivity

The compounding effects of hazards and anthropogenic drivers (Fig. 2) have impacts on the ecological sensitivity. This can be measured with data on the quantity and quality of “freshwater released downstream” (SDG 6.4.2), through the analysis of the “river and wetland disconnectivity” and the habitat degradation (fragmentation or destruction) (ES_FRA1) (Fig. 3).

##### Degradation of mangroves and other forests

Stakeholders and communities raised the issue of the erosion of the coastline, threatening mangrove forests. Indeed, coastal river deltas may lack the sediment supplies necessary to maintain their morphology (Das and Vincent 2009; Brammer 2014; Auerbach et al. 2015; Das 2020; Lázár et al. 2020). This impact arises from the retention of fresh water, alluvium and sediment by lateral (dams) and longitudinal engineering infrastructures (sea walls, dikes and embankments, roads). This infrastructure alters the flow of water (and its connectivity) and impacts riverine ecosystem functionality (Kuenzer et al. 2013). The status of the forest with its loss and gains resulting from the degradation or restoration and deforestation or reforestation is assessed with the SDG indicator “Forest area as a proportion of total land area” (SDG 15.1.1) and can be monitored with the use of satellite images. The impact of the infrastructures on the biodiversity can be measured by the indicator “Species richness” (SDG 15.5.1) assessed by the IUCN Red list Index (Islam et al. 2016). Moreover, exotic species are introduced in the mangroves and in shrimp farms and need to be monitored in the long run to assess the potential damages caused by their potential invasiveness (SDG 15.8.1) (Biswas et al. 2018). This can contribute to the loss of habitats and the loss of biodiversity. Therefore, assessing the forest cover (SDG 15.1.1) and the mangrove forest evolution (increase/decrease) informs on the state of biodiversity and on the ecosystem sensitivity (Veettil et al. 2019a).

##### Loss of ecosystem services

Communities benefit directly and indirectly from diverse types of habitats such as mangroves that provide many regulating ecosystem services such as the attenuation of storm surge and salinity intrusion, or the absorption of waves by mangroves that diminish the impacts of coastal erosion. Communities also benefit from provisioning ecosystems services including honey, firewood, fodder and access to aquatic organisms. Thus, when these ecosystems are affected by hazards, populations also lose their access to resources. According to village heads and heads of the Women’s Union and Youth Union of the Red River delta, the degradation and the fragmentation of ecosystems result in part from the use of fishing methods that can damage the mangroves, as well as from the unsustainable extraction of fish and sea products along the coast and in the river network **(**Fig. 3**)**.

##### Land conversion and habitat fragmentation, degradation and destruction

The river delta regions have been exporting rice (paddy) for the global market since the colonial times. Rice intensification has increased pressure on soils due to multiple cropping, with two to three crops on the same land unit per year (Freed et al. 2020). As a result, increased production ensured better food security (SDG 2), but at the same time, the use of fertilisers and pesticides to grow high-yielding varieties negatively impacts soil structure and composition. Moreover, since the 1990s, demand in seafood from the global market is increasing and requires the development of the agro-industry. Rice cropping has been replaced with shrimp aquaculture in delta coastal areas on land where the productivity is already declining due to increased soil salinity. As reported by communities and stakeholders in the Mekong and the Red River deltas, in all three river’s mega-deltas, land conversion is a main driver of delta ecosystems fragmentation, degradation and destruction.

##### Soil and water degradation

Results from focus group discussions and key informant interviews show that in aquaculture production, the use of aquafeeds and veterinary drugs to raise the shrimp increases water pollution. The change from rice cropping to aquaculture can become irreversible as soils become ever more saline. When possible, farmers prefer to keep the rice–shrimp farming as this helps to maintain a better water and soil quality (Nguyen 2015; Tyagi and Sen 2019; Braun et al. 2019; Nguyen et al. 2020; Thakur et al. 2021). As soil quality is of major concern for all stakeholders, an indicator was selected to measure the “Soil organic matter” and the “Area covered by problem soil”. This is not available in the SDG or in the SFDRR, even if it can be linked with the indicator “Proportion of land that is degraded over total land area” (SDG 15.3.1).

The factories of the small and medium industrial zones and urban areas all release pollutants to the canals (arsenic, fluorides, nutrient loads, antibiotic and agrochemical residues) (Binh et al. 2018; Braun et al. 2019). Coastal water quality can be measured through indicators of “Coastal eutrophication and floating plastic debris density” (SDG 14.1.1), the “Proportion of domestic and industrial wastewater flows safely treated” (SDG 6.3.1) and the “Average marine acidity” (SDG 14.3.1).

#### Lack of ecosystem robustness

##### Lack of policies to support the conservation of biodiversity

Policies can support the robustness of ecosystems through incentives aiming at strengthening the conservation, protection, or restoration of ecosystems within important sites for coastal biodiversity. This can be monitored with the indicator on the “Proportion of national exclusive economic zones managed using ecosystem-based approaches” (SDG 14.2.1) and the “Coverage by protected area of important sites for coastal biodiversity” (SDG 14.5.1). We found that efforts were made in all deltas to restore mangrove, with the creation of co-management committees in the Mekong Delta for example, to ensure the protection of replanted areas. This can be assessed with the indicator on “Progress toward sustainable forest management” (SDG 15.2.1). Moreover, different support from Official Development Assistance funds and public expenditure can be given for the conservation and sustainable use of biodiversity and ecosystems (SDG 15.a.1); this is considered as an asset in the implementation of policies (Wolf et al. 2022). However, the extension of aquaculture in the coastal areas and of clam farming in the mudflats creates a major trade-off for the sustainable use of terrestrial and inland freshwater ecosystems and their services (SDG 15.1.2) with a potential impact on the strategic plans for biodiversity (SDG 15.9.1).

##### Lack of enforcement of policies to protect ecosystems

Communities that earn incomes from tourism are also affected by the loss of recreation areas provided by the mangrove forest (Ghosh and Ghosh 2019; Chakraborty et al. 2020). At the same time, smuggling and poaching (SDG 15.7.1) is still reported in the mangrove forests of all the deltas (Duffy et al. 2016; Fig. 3). World views are changing with the loss of forest animals, which inspires spiritual values to be used as an argument to support the enforcement of policies to protect ecosystems (Jalais 2014).

#### Social susceptibility

##### Poverty and economic inequalities

During the consultations, indicators such as the “Population living below the international poverty line” (SDG 1.1.1) and below the “National poverty lines” (SDG 1.2.1), as well as the “Proportion of men, women and children of all ages living in poverty in all its dimensions according to national definitions” (SDG 1.2.2), the “Social protection systems for all types of deprived populations” (SDG 1.3.1), the “Access to basic services” (SDG 1.4.1) and the “People living below the median income” (SDG 10.2.1) were selected as indicators to assess the “Social susceptibility” in coastal areas.

##### Livelihood dependency on agricultural economy

Several indicators have been identified to assess the risk to livelihood in relation to the agricultural economy. This can be assessed with data on the “Volume of production per labour unit by classes of farming, pastoral or forestry enterprise size” (SDG 2.3.1), and the “Average income of small-scale food producers” (SDG 2.3.2) that can be threatened by the disasters.

Global trade and national markets involve pressures on coastal areas' natural resources and on the labour force to produce crops for exportation. The increasing economic demand for crops and seafood potentially encourages intensive cropping and monoculture. In case of crop failure, the rural population is the most vulnerable to food prices anomalies and volatility (SDG 2.c.1). Local agriculture production remains an important safety net to ensure food security as it compensates for the lack of access to market products (George and McKay 2019). Additionally, it is important to measure the prevalence of undernourishment (SDG 2.1.1) and the prevalence of food insecurity in the population (2.1.2).

##### Access to land ownership

The loss of land due to disasters (coastal or river erosion) and the inequitable access to resources creates marginalisation and the loss of small farmers’ sustainable livelihoods (SDG10)**.** Therefore, livelihood sustainability needs to be assessed through the access of the population to secure tenure rights to land (SDG 1.4.2), with legally recognised documentation.

Nevertheless, securing tenure rights is not always in favour of all the users of the forests and the mudflats managed as a village’s common property and, thus, as a public land. As reported by the communities of the Red River delta, the privatisation of the inter-tidal mudflats for clam farming has led to the likelihood of loss of livelihoods of local communities, especially of the poor, female heads of households and women who used to collect the seafood products in the common areas. Therefore, it is important to assess “women’s equal right over agricultural land” (SDG 5.a.1) and access to “land ownership and/or control” (SDG 5.a.2) (UN Women 2020).

Additional indicators are needed for the GDRI, to measure the land distribution among the farmers. Small farmers can own 1.5–5 ha, while large seafood businesses and companies hold 20–100 ha for intensive and super-intensive agro-industrial aquaculture (Sohel and Ullah 2012; Smith et al. 2013; Loucks 2019). Farmers have also unequal access to the supply chain infrastructure, as the produces are collected from the coast and then processed in the agro-industrial zones to be exported to the national and the international markets.

##### Safe waters and health

Human health includes a selection of indicators addressing, e.g. waterborne diseases or arsenic in drinking water (SDG 6). The GBM—and to a lesser extent also the MRD—is highly exposed to arsenic-contaminated groundwater and all delta populations are highly exposed to polluted surface water (Agusa et al. 2009; Ahmad et al. 2018). Natural hazards may also reduce the capacity of the water supply system to continue delivering services during and in the aftermath of (potential) hazards and for people to have an equitable access to safe and affordable drinking water services (SDG 6.1.1). This is also the case for the access to adequate and equitable sanitation and hygiene services (SDG 6.2.1). Therefore, the population needs to have access to a sewage drainage system and to a good ambient water quality of water bodies (SDG 6.3.2).

The pollution of river delta channels and of aquacultural ponds has a major impact on the quality of farmed products and on its quantity. Contamination by bacteria frequently cause death of black tiger shrimp, white-leg shrimp, clams, fish and crabs. Moreover, exotic invasive species sometimes take over the ponds (SDG 15.8.1). Damage caused by pollution can have major impacts on farmers’ productions and, thus, on their income.

##### Migrations

Due to poor harvests and crop failures and depending on their access to land ownership with secure tenure rights (SDG 1.4.2) and on employment rates in rural areas, many households have abandoned agricultural production and moved to the cities, i.e. Can Tho, Ho Chi Minh City, Dhaka, Kolkata and Hanoi in the studied deltas or in their vicinities. To assess this dynamic, it is important to look at the rates of migration from rural to urban areas (SDG11). The new urban populations are most often settling in old urban centres or on the outskirts, where they take shelter in unsafe informal settlements, slums, or inadequate housing (SDG 11.1.1). In those settlements, households and migrants face multiple challenges that would benefit from the implementation of planned and well-managed migration policies (10.7.2).

##### Access to services

It is important to measure the proportion of populations that have access to public transport (SDG 11.2.1) and public and critical infrastructures such as schools, hospitals or private markets. In both urban and rural settlements, populations still have unequal access to key economic sectors and services; water (SDG 6) and energy supply (SDG7), transportation infrastructure (SDG 9) and construction and housing (SDG 11). The lack of access to basic services have clear implications for the susceptibility of the SES.

#### Lack of coping and adaptative capacity

To assess the risk to social–ecological systems, experts, stakeholders and communities were invited to identify the root causes for the lack of coping and adaptive capacities in different sectors: agricultural systems, urban development, disaster risk management and governance systems.

##### Lack of adaptive agricultural systems

Adaptation and coping strategies depend in part on the use of a diversified set of agricultural systems that can adapt to different potential hazards. In the long run, it goes with the implementation of a sustainable food production system. This can be measured with the “proportion of agricultural area under productive and sustainable agriculture” (SDG 2.4.1). These are sustainable agricultural practices that increase productivity and production and help maintain ecosystems, strengthen capacity for adaptation to climate change, extreme weather, drought, flooding and other hazards and progressively improve land and soil quality (SDG 2.4).

Different varieties of plants can adapt to the river and coastal dynamics: floating rice can adjust to variation of flood water levels; other varieties can be salt or drought tolerant. Therefore, maintaining the genetic diversity of seeds can support the adaptation capacities (Nguyen 2015; Nguyen et al. 2019, 2020; Tu Nguyen et al. 2019; van Kien et al. 2020; Nguyen Thanh et al. 2020; Haque et al. 2021). This can be measured by the “number of plant and animal genetic resources for food and agriculture secured in either medium- or long-term conservation facilities” (SDG 2.5.1), as well as with the monitoring of the use of “local breeds classified as being at risk, not at risk or at the unknown level of risk of extinction” (SDG 2.5.2). It informs on the risk of loss of local varieties traditionally used by farmers to adapt to uneven climatic conditions. However, many rice varieties have been lost due to agricultural intensification and monocropping.

These indicators can be completed with information on the “loss of language diversity” (endangered languages) and the consequent potential “loss of local ecological knowledge”. Indeed, recognition, protection and promotion of indigenous and local knowledge can strengthen economic, environmental, social and cultural resilience within societies and form the knowledge base for addressing critical sustainability problems (UNSAB 2016).

##### Lack of health infrastructures

The coverage and accessibility to essential health-care services (3.8.1) is important to assess the capability to prevent and treat injuries (SDG 3.d.1). The existence of an organised and functioning emergency relief facilitates coping during cyclones or flood events (SDG 3.d.1). This can be measured through the density of emergency services: hospitals, fire brigades and police stations. Access to health-care services includes the availability of sufficient beds in hospital (SDG 3.8.1) to cope with disaster events as seen during the COVID-19 pandemic.

##### Lack of infrastructures

The availability of individual or collective means of transportation (SDG 1.4.1) and the access to tele-communications is necessary to contact emergency services, and reach cyclonic shelters. Public infrastructures, constructed on high stilts, can reduce population’s the exposure to cyclones, floods and tidal surges. Individual locally made houses (e.g. kutcha houses made of bamboo, mud, palms and/or straws) may not have the strength to withstand these hazards. Therefore, social capacity is not only a consequence of the physical location but also depends on the infrastructures available.

##### Lack of early warning systems

The response may depend on the early warning systems based on the monitoring of hazards. The early interpretation of information and signals, as well as actions to take in case of damage caused by hazards, can be supported by disaster preparedness training of communities and local administration. Both the GDRI and the SFDRR contain an indicator on the availability of/and access to multi-hazard early warning systems (G1–G6 of the SFDRR) (which the SDGs do not). But only the GDRI contains indicators for access/availability to emergency services (e.g. hospitals, fire brigades and police stations).

##### Lack of awareness and preparedness

The consultation has also highlighted the importance of raising awareness and improving preparedness through effective dissemination and preparation of response in case of events that can affect rural or urban areas (SFDRR, SDG 1.5.3—SDG 11.b.1–2). The efficiency of awareness and preparedness tools is also interconnected with the literacy level of the population and the education of all genders to global citizenship (SDG 4.6.1, 4.1.1 and 4.7.1). Moreover, the participation in trainings (SDG 4.3.1) is also important to transmit the skills for the use of information and communication technology (SDG 4.4.1) and, thus, ensure the capacities to monitor the ecosystem sensitivity.

##### Lack of disaster risk reduction strategies and planning

Adaptive and coping capacities can be enhanced by the planning of disaster risk reduction strategies at different scales. This is tackled by target E of the SFDRR “Substantially increase the number of countries with national and local disaster risk reduction strategies by 2020”. This target is monitored by all the frameworks with the indicator: “Percentage of local governments that adopt and implement local disaster risk reduction strategies in line with national disaster risk reduction strategies” (SDG 1.5.4, 11.b.2 and 13.1.3 and SFDRR E). Doing so, disaster management committees are mandated to prepare the local response plans. These are reported for the National Adaptation Plans. These can include actions such as consolidation of infrastructures (in case of breaches in embankments) or reinforcement of evacuation roads. Strengthening the capacity building of institutions and individuals would support this implementation of adaptation, mitigation and transfer of technology (SDG 13.3.2).

### Social and ecological exposure

The Mekong, Red River, GBM-I and GBM-B deltas are densely populated spaces with an average density of 600 inhabitants/ km^2^ in rural areas (Szabo et al. 2016a, b; Edmonds et al. 2020). The population is still increasing despite the rates of growth having slowed since 2010 in rural areas. The SFDRR indicators measure social exposure of populations and infrastructures exposed to hazards. Assessing the “number of deaths” or “injury” (SDG 1.5.1, 11.5.1 and 13.1.1 and SFDRR A-1 to A-5) or “damage on dwellings” or “destroyed dwellings” attributed to disasters (SFDRR B-3 and B-4) is of major concern in all the deltas. The population growth is concentrated in urban areas. Unplanned urban sprawling sometimes involves constructions in the most exposed to floods areas and can cause injuries and death (SDG 11.5.1, SFDRR A). Thus, populations—whether urban or rural—are differently exposed to hazards depending on the location of their settlements, whether along the coast or inland, along the main river channels or along smaller streams and canals.

#### Exposure of critical infrastructure

‘Critical infrastructure’ is a term used in the SFDRR and by governments to describe assets essential for the functioning of a society and economy. It includes infrastructures to produce energy, education, health, transport, digital communications, water and sanitation, as well as the infrastructure essential to protect them, such as bridges, roads, seawalls, embankments or dikes and canals. It is cited as an integral component of the SFDRR indicators Target D “Damage to critical infrastructure attributed to disasters” and by SDG indicator “11.5.2 Direct economic loss in relation to global GDP, damage to critical infrastructure and number of disruptions to basic services, attributed to disasters”. All these infrastructures are unequally exposed to hazards, depending on the strength of the architecture, the condition of buildings and the materials used. For example, anticyclonic shelters are generally built to support the most extreme events, while other infrastructures such as schools and hospitals are not constructed to withstand the same level of hazard.

Embankments, dikes, seawalls, polders and other hydraulic infrastructures are widely constructed across the deltas to protect delta populations. However, these often create the so-called “levee effect” (Ferdous et al. 2019) as a side effect of hydraulic infrastructures: they can defend and protect the coast and keep the people safe if they are well planned and maintained, but they can also create a false sense of protection and the damage can be even greater in case of breach (Brammer 2014; Auerbach et al. 2015; Ishtiaque et al. 2017; Hussain et al. 2018; Knox et al. 2022). Thus, as reported by experts, stakeholders and communities in this study, breaches in embankments or sea walls due to cyclones Amphan and Yaas have caused sudden freshwater floods and saline water intrusion (Ghosh and Mistri 2020, 2021; Chaudhuri et al. 2022). Besides short-term disturbance, these events can cause long-term waterlogging if the water cannot be drained out through sluice gates (Gain et al. 2019).

#### Exposure of non-cultivated ecosystems

Deltas' waterscapes are composed of sensitive ecosystems (mangrove forests, mudflats, lakes, swamps and lagoons and others) exposed to hazards and to human pressure in many ways. Unless transformed and controlled by human interventions, river delta waterscapes are constantly reshaped by the forces of the river and tidal water that shape floodplains, wetlands, river channels and mudflats. The patchy transitions between land, fresh, brackish and saline water depend on the tidal saltwater intrusions and the freshwater discharge from the river (Chen and Mueller 2018; Ahmed et al. 2018; Nguyen et al. 2019; Sherin et al. 2020; Das et al. 2020b). These dynamics generate diverse habitats and high biodiversity, in turn providing a multitude of ecosystem services to communities (Nicholls et al. 2018a, b; Akter et al. 2019; Lázár et al. 2020).

Mangroves are important ecosystems, as they capture the sediments and regulate coastal floods, erosion and saline water intrusion. A part of these ecosystems is included in protected areas implemented to conserve heritage sites and endangered species. However, these ecosystems are shrinking due to coastal erosion, deforestation and pollutions produced by intensive aquaculture and rapid agro-industrialisation (Bui et al. 2014; Loucks 2019).

Non-cultivated ecosystems are not captured by SFDRR indicators. However, we could link the ecosystems to the Target C “loss in relation to GDP” if we consider the economic benefits received from ecosystem services (Farber et al. 2002; Hossain et al. 2017; Adams et al. 2018, 2020; Chakraborty et al. 2020). The role of mangrove in contributing to the regional economy is increasingly recognised (Phan and Stive 2022). The exposure of ecosystems is inherent across SDGs, for example, the SDG indicator “6.6.1 Change in the extent of water-related ecosystems over time”. Without mangroves, agro-ecosystems and settlements along the coastline are directly exposed to multiple hazards*^3^ (Das and Vincent 2009; Zhang et al. 2012; Deb and Ferreira 2017; Veettil et al. 2019a, b; Das 2020a, b, c; Phan and Stive 2022).

#### Exposure of agro-ecosystems

Several agro-ecosystems are present in deltaic coastal areas. The distribution is similar in all three mega-deltas (Nguyen et al. 2019; Aravindakshan et al. 2020; Yuen et al. 2021; Pham et al. 2021). From the coast to further inland, farming systems are based on:

1. Seafood collection or farming (including clams) in the mudflat.
2. Crab or shrimp aquaculture systems for two main species: white-leg shrimp (*Penaeus vannamei*) and the black tiger shrimp (*Penaeus monodon*). These include both mangrove–shrimp farms, the extensive, intensive and super-intensive shrimp farms. This farming system is in the brackish-water zone, where salinity values higher than 0.4 ppt can be found all year round (Haigh et al. 2016; Nguyen et al. 2019, 2020).
3. Rice–shrimp or rice–fish rotation systems are practised further inland, in the brackish-water zone. In these systems, rice is cultivated during the rainy season, when fresh water dominates for 4–6 months. That same land is then switched to shrimp farming during the dry season when the salinity of the water increases (Nguyen 2015; Tuu et al. 2020; van Kien et al. 2020; Maitah et al. 2020; Freed et al. 2020; Nhat Lam Duyen et al. 2021).
4. The double-rice system (two crop seasons per year) or land characterised by the rotation of rice and other annual crops is found in the freshwater zone, upstream of the delta (Nguyen Thanh et al. 2020; Pham et al. 2021).
5. Livestock (Aravindakshan et al. 2020).
6. Orchard and vegetable gardening (Nguyen et al. 2015; Renaud et al. 2015; Veettil et al. 2019a).

Despite the rapid urbanisation and industrialisation, the agricultural sector is still an important source of livelihood for billions of people in the deltas. The employment rate in agricultural employment has declined in Bangladesh from 59.9% in 2002 to 39.4% in 2018, 58.6% in 2002 to 43.3% in India and 62% in 2002 to 38.4% in Vietnam.^4^ Agricultural sector’s contribution to the economy still adds up to 14.9% of the gross domestic product (GDP) for Vietnam^5^ (Maitah et al. 2020), while the contribution is declining in Bangladesh from 17% in 2010 to 12% of the GDP in 2020 and increasing in India from 17.2 to 18.2% of the GDP. Therefore, impacts of hazards on agricultural production may involve loss of GDP (SDG 1.5.2). This is assessed by the SFDRR indicator (B-5): “Number of people whose livelihoods were disrupted or destroyed, attributed to disasters”.

### Alignment of the GDRI with the SDG and SFDRR frameworks

**Fig. 5:**
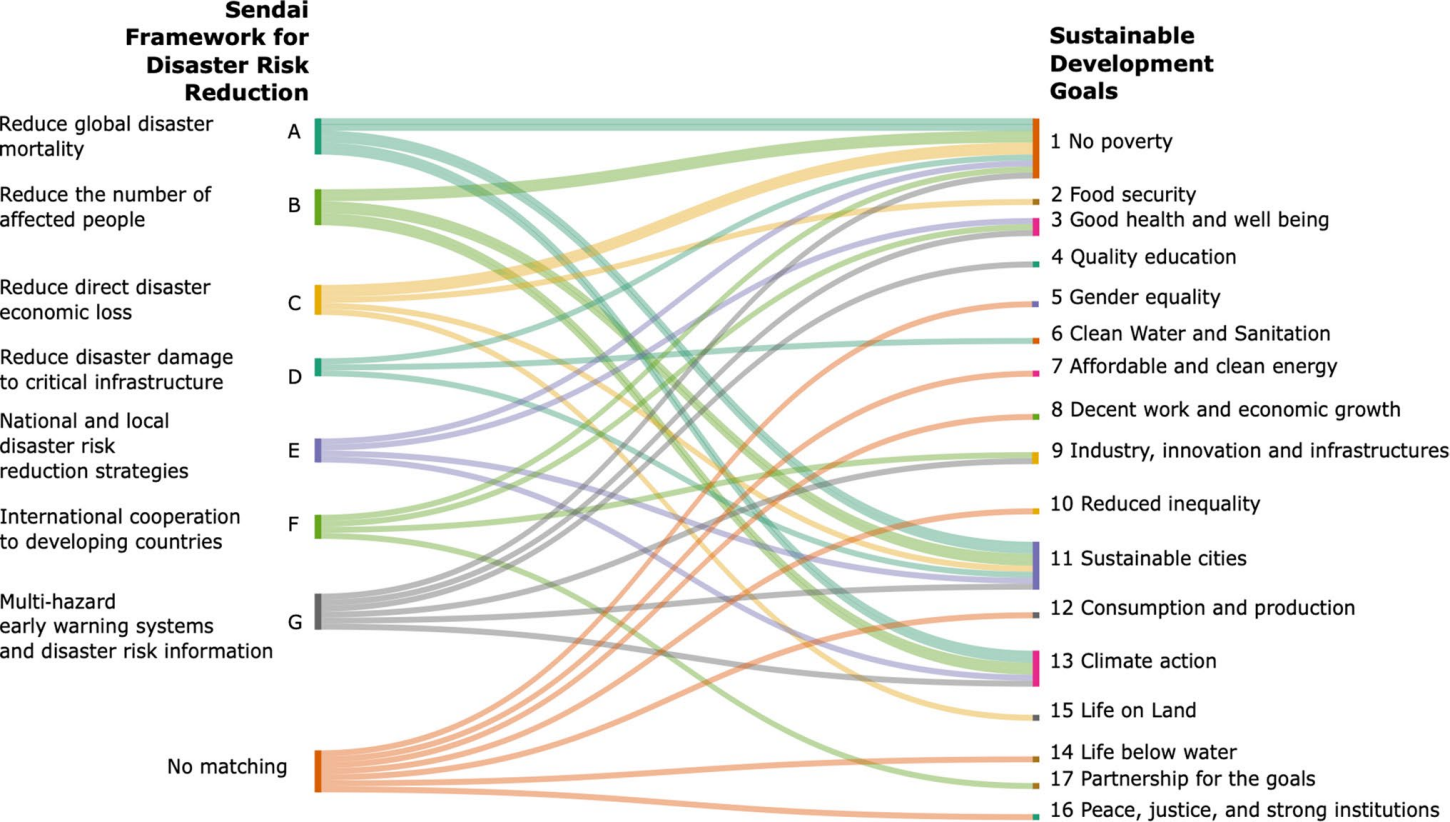
Alignments between the SFDRR and SDG framework. Source: Sankey plot of SFDRR targets and SDGs. Designed by Emilie Cremin, 2022

In the context of our three river deltas, 83 SDG indicators across the 17 goals were identified during the consultation and analysed through the deltas expert's workshop. It appears, after comparing the list of indicators, that 18 indicators selected are used in both frameworks across the 7 SFDRR targets and 9 SDGs (Fig. 5).

The SFDRR indicators focus mainly on SDGs 1, 11 and 13. The SFDRR also provides indicators measuring the impact of disasters with more accuracy than the SDGs regarding the level of loss, damage and destruction. However, the Sendai Framework does not cover the targets of SDGs 5, 7, 8, 10, 12, 14 and 16.

The indicators of these global frameworks are designed for international comparisons and are mainly measured at a national level. There is a need for governments and regional authorities to assess risks at various administrative scales to better address issues of territorial exposures and vulnerabilities.

The SDG/GDRI matrix (Table 2) highlights the matching and the number of indicators selected per SDG and per GDRI component. The SDG indicators selected matched primarily with the “social susceptibility” (29 indicators) and the “lack of coping capacities and adaptation strategies” (30 indicators) components of the GDRI, across 16 of the 17 SDGs. This reflects the focus of SDGs on social issues.

Ten SDG indicators matching the “Ecosystem robustness” and 6 SDG indicators matching the “Ecosystem sensitivity” were selected among the SDGs 6 (Clean water and sanitation), 14 (Life below water) and 15 (Life on land). This shows the extent of the ecological issues considered in the SDGs. For instance, ecosystems provide services regulating and reducing the impact of hazards that can be measured using SDG indicator 15.1.2 (Proportion of important sites for terrestrial and freshwater biodiversity that are covered by protected areas, by ecosystem type). The GDRI provides 28 additional indicators to better assess ecosystem exposure, sensitivity and robustness.

Only five SDG indicators selected could be matched with the “Social exposure” component of the GDRI, but none with “ecosystem exposure”. The highest number of matching indicators were found in the SDG 1 (No poverty) with 12 indicators selected and in the SDG 2 (Zero hunger) with 10 indicators selected. This highlights the specificities of the deltas studied as major areas of agricultural production ensuring food security.

A smaller number of indicators were selected among the SDG 7 (Affordable and clean energy), SDG 8 (Decent work and economic growth), SDG 12 (Sustainable consumption and production), and SDG 17 (Partnership for the goals). These topics were discussed less frequently during the consultations, reflecting the areas of expertise of the people interviewed and the potential trade-offs regarding these SDGs (Kroll et al. 2019).

It is worth noting that SDG 5 (Gender equality) only matched with 2 indicators selected. This may reflect the gender bias of the consultation as presented in the methods section. The lack of gender indicators represents a more systematic issue in risk frameworks beyond the consultation group.

The SDG/SFDRR matrix (Table 3) highlights the matching and the number of indicators selected per SFDRR target and per GDRI component. The SFDRR indicators selected matched mainly with the “social susceptibility” (5 indicators), “lack of coping capacities and adaptation strategies” (10 indicators) and the “social exposure” (12 indicators) components of the GDRI. This reflects the focus of SFDRR on adaptation strategies and on social exposure.

However, only one SFDRR indicator could be matched to the “ecosystem sensitivity” component and none could be matched either to the “ecosystem sensitivity” or to the “ecosystem exposure” components of the GDRI. On the one hand, this illustrates the gap of the SFDRR in considering, at the indicator level, the impact of hazards on ecosystems, as global risk assessment frameworks do not address well the root causes of the risk and the ecological vulnerability is often under-evaluated (see Marin-Ferrer et al. 2017). On the other hand, it shows the GDRI emphasis on social–ecological systems.

The GDRI aims at being used to assess the risk at the regional or delta scale and down to the local scale (administrative levels 2 and 3). Therefore, some meaningful indicators of the SDGs and the SFDRR were not kept, when the scale was only applicable to the national or international scale, unless the indicators could inform on the policies that can have impacts at the local level.

Given the reuse of some indicators in different targets of the SDGs and some overlap with the SFDRR indicators, the resulting version of the GDRI library (S4) encompasses 143 indicators, out of which 80 are aligned with the SDG indicators, SFDRR, or both. This represents an alignment of nearly 56% of the GDRI indicators with these two frameworks.

## Conclusion

This paper is primarily the result of a consultation with regional delta experts, key informant interviews, focus group discussions with communities and workshops with stakeholders in the Red River, Mekong and GBM (Ganges–Brahmaputra–Meghna) river deltas, in Vietnam, India and Bangladesh. This consultation provided opportunities to integrate local and scientific knowledge in an analysis of the root causes, the cascading processes and the compound effects of hazards. The analysis of the results shows the complexity of the interconnections between hazards and anthropogenic drivers of change and their impacts on the vulnerability of social–ecological systems. The impact chain we have produced and the indicators we have selected consider the links between social systems and ecological systems as key to understand the causes of ecological threats in the deltas.

The comparative analysis of the SDG and the SFDRR frameworks with the selected indicators reveals that these global frameworks do not capture well the ecological impacts of hazards. Indeed, the SDG framework only provides a few indicators related to ecological sensitivity and robustness, while the SFDRR does not have any. Through this process, we provide an updated library with 28 additional indicators to better assess ecosystem exposure, sensitivity and robustness.

Further integration of policy and monitoring infrastructure between these frameworks could help to address issues of capacity, governance priorities and data availability. Maximising the relevance of specific monitoring agencies across different frameworks and cross-cutting of indicator data could help to enhance data availability and address the issues of data scarcity that hinder the SDG process, particularly at the local level.

This research also contributes to addressing some of the limitations of the SDGs and the SFDRR frameworks designed at a global scale. While some indicators assessing the progress towards the goals of those frameworks can be integrated in a risk assessment, other indicators referring to the national or international scale do not provide an in-depth assessment of local issues. The GDRI helps to bridge this gap and is designed to make a risk assessment at a sub-delta level. It provides more details on local, social and ecological vulnerability than the global frameworks considered. Indeed, it informs not only on the number of people affected by disasters, but more importantly on why and how they are affected. This level of specificity is critical for sustainable development, particularly in the deltas’ SESs.

This qualitative analysis is the basis for future work regarding the computation of the GDRI with the updated library of indicators. Based on experts and stakeholder consultations, the tailored tool can be used to improve the information produced for policy makers and communities. The GDRI aims at helping to prioritise the interventions over the deltas with an increased accuracy on the most at-risk areas. This tool will support stakeholders in their effort to co-develop strategies aiming at reducing communities' and ecosystem's exposure to hazards through an improved disaster risk reduction planning and will further help to better prepare, respond, recover and build back better when ever hazards strikes again over delta's social-ecological systems.

## Author contributions 

Conceptualisation: EC. Methodology: EC, FGR, ZS, JO’C. Formal analysis and investigation: EC. Writing—original draft preparation: EC. Writing: EC. Review and editing: EC, SB, LHB, AC, HHH, DVH, HL, SM, SM, JO’C, AV, ZS, AL, FGR. Funding acquisition: AL. Data collection: EC, SB, LHB, AC, HHH, DVH, HL, SM, SM. Supervision: FGR.

## Supplementary Information

Below is the link to the electronic supplementary material.

S1. Definition Box for the words referred with an * in the text and S2. General methodological flow (DOCX 28 KB)

S3. The Impact chains: The impact chain will be provided in a PNG file (PDF 1133 KB)

S4. Detailed list of indicators: Excel file with details of the GDRI’s components, sub-components with description, categories and indicators (XLSX 480 KB)

## Data Availability

The datasets generated and analyzed during the current study are available from the corresponding author on reasonable request.

## Footnotes

1 https://livingdeltas.org/ available on the 25th of May 2022.

2 See detailed list of dams on the World commission on dams website: https://www.icold-cigb.org/GB/world_register/database_presentation.asp

3 the words with an * are defined in the Electronic Supplementary Materials (ESM) 1 (S1).

4 World Bank: http://wdi.worldbank.org/table/3.2

5 World Bank: http://wdi.worldbank.org/table/4.1
