# Supplementary material for: Aligning the Global Delta Risk Index with SDG and SFDRR global frameworks to assess risk to socio-ecological systems in river deltas
Source: Sustain Sci. 2023 Mar 3:1–21. Online ahead of print. doi: 10.1007/s11625-023-01295-3 (PMC9982774; doi:10.1007/s11625-023-01295-3)
Supplement: Supplementary file 1 — S1. Definition Box for the words referred with an * in the text and S2. General methodological flow (DOCX 28 KB) [file 11625_2023_1295_MOESM1_ESM.docx]

# Aligning the Global Disaster Risk Index with the Sustainable Development Goals to assess risks to socio-ecological systems in river deltas

# Supplementary materials

## SM1. Definition Box for the words referred to with an * in the text – key words are defined here in the alphabetic order

**Adaptation** is defined in human systems as the process of adjusting to actual or expected climate and its effects to moderate harm or take advantage of beneficial opportunities. In natural systems, adaptation is the process of adjustment to the actual climate and its effects; human intervention may facilitate this.

**Alignment** is the process of identifying synergies among policy processes with common objectives to increase efficiency and effectiveness for improved outcomes.

**Disasters** are the result of hazards and their impacts.

**Ecosystem or ecological sensitivity or susceptibility** is defined as ecosystems' vulnerability to external and internal transformative processes such as habitat destruction, saline intrusion, subsidence, and land use changes. We use in this framework ecosystem “sensitivity” as an ecological terminology.

**Ecosystem or ecological robustness** is defined as the capacity of an ecosystem to recover from disruptions. This can be human support to habitat restoration (through ecological engineering, Nature based-solutions or other ecological-based adaptations), nature conservation policies (inclusive or exclusive human), agro-ecological projects (organic farming, etc.), regulatory policies (permits for natural resources collection, etc.).

**Exposure** is defined as the presence of people; livelihoods; species or ecosystems; environmental functions, services, and resources; infrastructure; or economic, social, or cultural assets in places and settings that could be adversely affected (IPCC, 2022). **Exposure of a socio-ecological system** can be characterised by its socioeconomic state and its ecosystem state. If population and economic resources were not located in (exposed to) potentially dangerous settings, no problem of disaster risk would exist (Birkmann et al., 2013; Cardona et al., 2012b; UNISDR, 2015).

**Hazard:** **Natural and Anthropogenic hazard** refers to the possible, future occurrence of natural or human-induced physical events that may have adverse effects on vulnerable and exposed elements (Birkmann et al., 2013; Cardona et al., 2012a; Renaud et al., 2010). Hazard is defined as the potential occurrence of a natural or human-induced physical event or trend that may cause loss of life, injury, or other health impacts, as well as damage and loss to property, infrastructure, livelihoods, service provision, ecosystems, and environmental resources. Physical climate conditions that may be associated with hazards are assessed in Working Group I as climatic impact drivers.

**Multi-hazard:** According to UNDRR, the multi-hazard concept refers to “(1) the selection of multiple major hazards that the country faces, and (2) the specific contexts where hazardous events may occur simultaneously, cascading or cumulatively over time, and taking into account the potential interrelated effects.” Available on: <https://www.undrr.org/terminology/hazard>

**Resilience** is defined as the capacity of social, economic and ecosystems to cope with a hazardous event or trend or disturbance, responding or reorganising in ways that maintain their essential function, identity, and structure as well as biodiversity in the case of ecosystems while also maintaining the capacity for adaptation, learning and transformation. Resilience is a positive attribute when it maintains such a capacity for adaptation, learning, and/or transformation. **Resilience** is the capacity of a system to recover from stresses through regenerative processes. If the SES is confronted with a major change in the system's parameters, it may reach a **tipping point** (T. M. Lenton et al., 2012; Timothy M. Lenton, 2011; Renaud et al., 2013). Early warning signals would inform these tipping points (Timothy M. Lenton, 2020; Cardona et al., 2012). As a system approaches a threshold Early warning signals may show signs of growing instability, such as increased variability, skewness and autocorrelation linked to theories of critical slowing down and flickering (Dakos et al., 2008; J. A. Dearing et al., 2014; Timothy M. Lenton, 2020; Scheffer et al., 2012).

**Risk** is “the potential for adverse consequences” that can threaten livelihoods (IPCC, 2014; Oppenheimer et al., 2014; IPCC 2022; Reisinger et al., 2020) resulting from the impact of multiple natural and anthropogenic hazards, on social and ecosystem exposure. Key risks have potentially severe adverse consequences for humans and social-ecological systems resulting from the interaction of climate-related hazards with vulnerabilities of societies and systems exposed (IPCC, 2022).

**Social susceptibility** or fragility (in disaster risk management) or sensitivity (in climate change adaptation) **derives from the historical and prevailing cultural, social, environmental, political, and economic contexts**. In this sense, **vulnerable groups are not only at risk because they are exposed to a hazard but also because of social inequities that involve marginality in everyday patterns of social interaction. This involves restrictions in the access and access to resources, needed for communities’ livelihoods** (Cardona et al., 2012). Social susceptibility results from social dynamics and politics involving inequalities, unfair distribution, and access to resources. We also consider that social and ecological vulnerability arises from environmental injustice (Walker, 2012). Hazards such as coastal and inland flood risk strike differently communities depending on their social and economic capital. Social inequity and disparity can be measured by the capacities and rights to access resources (natural or transformed). It must be seen also through the quality and quantity of resources after considering the impacts of human actions on the environment.

**Transformability** refers to the capacity to create fundamentally new systems when the existing system becomes untenable (Walker et al., 2004).

**Vulnerability** is the predisposition of the elements and processes of the SES to be adversely affected or the propensity or predisposition to be adversely affected and encompasses a variety of concepts and elements, including sensitivity or susceptibility to harm and lack of capacity to cope and adapt (IPCC, 2022). **Vulnerability** is linked to the **lack of coping and adaptation capacities**, related to the weakness of governance that involves incomplete **communication around risks**, especially the lack of appropriate information that can lead to false risk perceptions (Birkmann and Fernando, 2008), which have an important influence on the motivation and perceived ability to act or to adapt to climate change and environmental stressors (Grothmann and Patt, 2005). Moreover, **lack of coping and adaptation capacities** are also the results of maladaptation or unsustainable adaptation that can be associated with the lack of integration of knowledge (local and engineering) about hazards within the urban or rural planning that brings in weak infrastructures and housing architecture. Those infrastructures can get support from public policies, laws, and regulations. Nevertheless, the adaptation capacities are also related to access to services (transportation, health, water supply, housing, market, electricity, etc.) (Birkmann, 2013). It is characterised by the population (density, growth, age, gender, household size, etc.), livelihoods (sources of income, practices, landholding/ property), alternative income-generating activities, and diversification of crops. The education level associated with local knowledge supports awareness of risk and the capacity of a community to anticipate, respond and recover from a situation of disturbance and reorganize while undergoing change to maintain the same livelihood and identity.

## SM2. General workplan of the Living Deltas Risk Assessment Working Group

| **Stage 1. Revision of the GDRI conceptual framework for risk assessment**   - Revision of the GDRI conceptual framework – completed in November 2020 – see presentation of the RAWG - Review of literature and indicators applied in similar approaches => + comparison with SDG indicators - Review of Definition of risk to be tackled | Completed in October 2020  With additional revision ongoing |
| --- | --- |
| **Stage 2. Design of a generic Impact chain to inform the Global Delta Risk Index**  The GDRI Impact chain generic version is primarily developed by the Living Delta Hubs multidisciplinary team of researchers and delta experts. The Impact chains are designed for the risk assessment of 3 deltas: the GBM, Mekong, Red River.   1. Defining Delta’s Social-ecological systems – land uses and livelihoods - Identification of causes and effects of hazard, their impacts, and the vulnerability of the SES (Social susceptibility, ecological sensitivity, lack of adaptative capacities, Ecological Robustness, and policies) 2. Design of a generic version of the Impact chains based on literature review, SDG indicators and experts’ regional knowledge of the deltas (delta partners and colleagues). The IC generic version is presented to each delta teams of the LDH to create new impact chains detailing each delta specificities. 3. Delta specific impact chains are presented during workshops to the inhabitants of the delta during Focus Group Discussions, Key Informant Interviews and Stakeholder workshops, as a document for discussions, and susceptible of revisions and amendments. To complement the impact chain and spatialise the information, the participants were invited to a participatory mapping exercise. With the help of both documents, the participants could debate and refine the indicator list. (For this section see the document on stakeholder workshop organization) 4. The Delta-specific impact chains helps to define, select, and classify indicators. Based on the result of the previous activities, we have a large set of indicators compiled in the indicator library. This large list is shortlisted to have a selection relevant for each delta. At this point, we may have a list of a 100+ indicators. 5. Identification of valid indicators => List of prominent indicators => Indicator wish list 6. Outputs: journal papers and policy briefs | Completed  March 2022  1st submission in June 2022  Re-submitted in November 2022 |
| **Stage 3. Indicators selected ranked and scored by local stakeholders**  Once a revised list of indicators is defined, a questionnaire is submitted to delta’s local stakeholders. The selected indicator should be representative of all the delta coastal areas. It should include all types of livelihoods.   - **Organisation of stakeholder workshops to select and score the indicators** - **Selection of final indicator set** with a questionnaire - **Ranking of indicators** within each vulnerability sub-component | September 2022  and  January  2023 |
| **Stage 4. Survey and data collection**  Once the final set of indicators for the GDRI-c is deliberate, secondary data are collected (here we may find some proxy indicators, if we do not have the same data for all the deltas or if the data is not available for the targeted indicator). The data are analysed, and complementary data are collected if necessary. We look here for a good quality of data, representing, when possible, the lowest spatial scale (village or commune level).     - **Data collection – secondary data** - **Data collection – primary data - Field survey** - **Data acquisition** | 2022-2023 |
| **Stage 5. Implementation of a database – computation of risk**  The overall data set will be processed through the calculation of the results, normalization, and aggregation. The set of indicators can be combined and aggregated in categories to create composite indicators.   - **Calculation of the Index** - **Analysis and imputation of missing data / Detection and treatment of outlier** - **Assessment of multicollinearities** - **Normalization** - **Weighting** - **Aggregation** - **Final set of indicators** | 2023 |
| **Stage 6. GIS Visualisation – Geo-platform - for Risk assessment to be used by policy makers**    Finally, all the data are visualized in an GIS – if possible, on a GIS Geo-platform.  Output = Vulnerability and risk index | 2023 |
| **Stage 7. Outputs**  **Final publication in a journal** | 1st submission in June 2022  Re-submitted in November 2022 |
