# Supplementary material for: Aligning the Global Delta Risk Index with SDG and SFDRR global frameworks to assess risk to socio-ecological systems in river deltas
Source: Sustain Sci. 2023 Mar 3:1–21. Online ahead of print. doi: 10.1007/s11625-023-01295-3 (PMC9982774; doi:10.1007/s11625-023-01295-3)

Global Delta Risk Index - Impact Chain - Generic version

This impact chain is the result of a collaborative work within the UKRI GCRF Living Deltas Hub Grant : *NE/S008926/1*.
Activity coordinated by Emilie CREMIN from October 2021 to May 2022

The Global Delta Risk Index framework

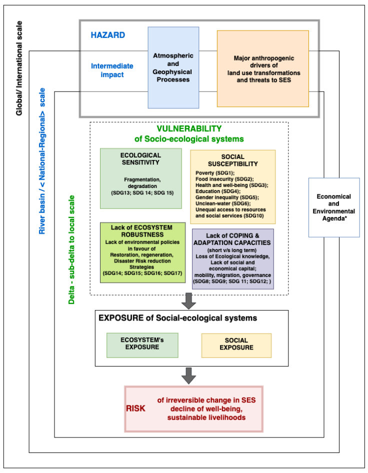

*SDG (Sustainable Development Goals) GBF (Global Biodiversity Framework), DRR (Disaster Risk reduction), PA (Paris agreement) Designed by Emilie CREMIN 2022, adapted from Sebesvari 2016, Gallopin 2006

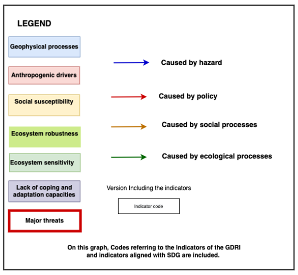

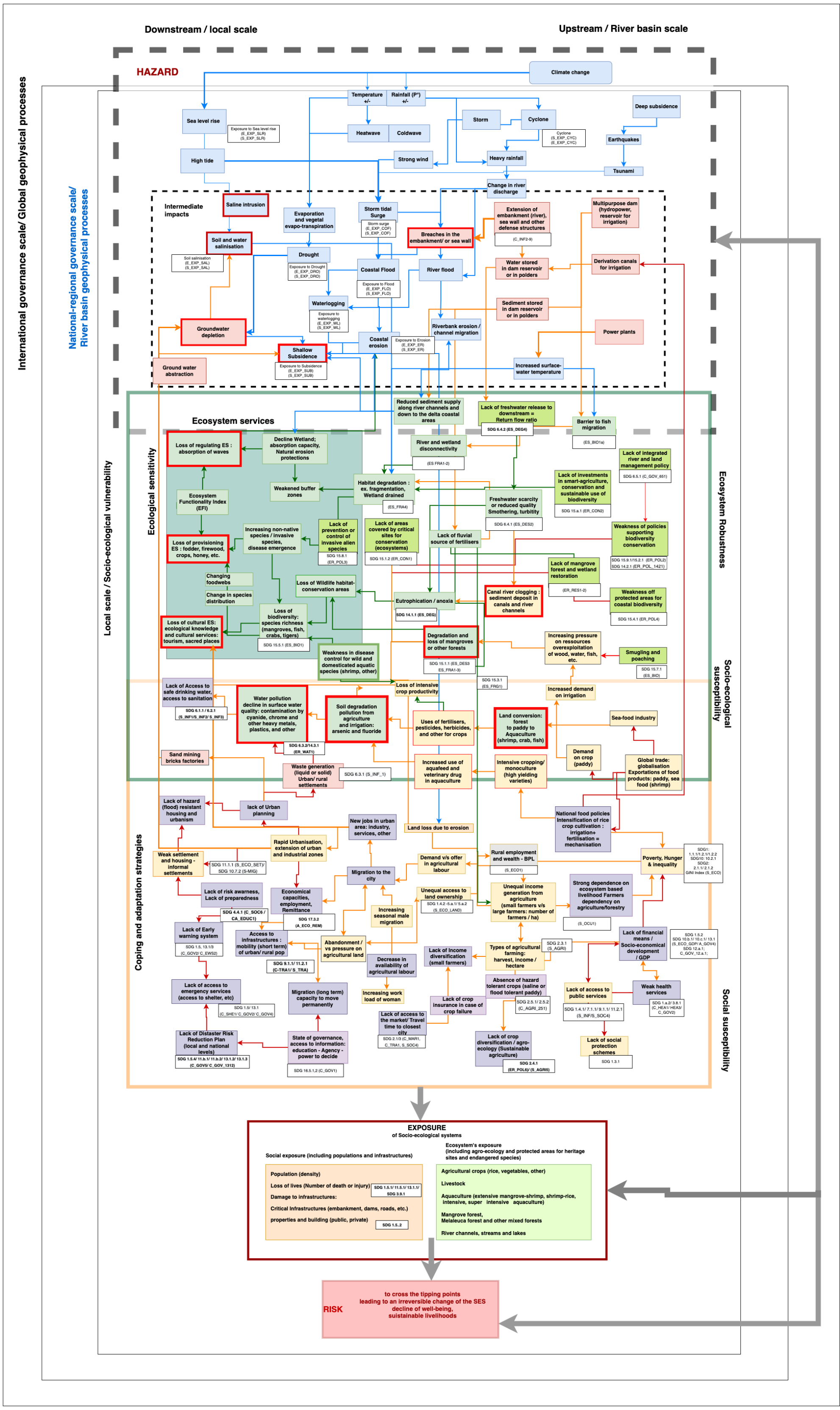

Supplement: Supplementary file 2 — S3. The Impact chains: The impact chain will be provided in a PNG file (PDF 1133 KB) [file 11625_2023_1295_MOESM2_ESM.pdf]
